# Virulence Factors and Antibiotic Resistance of *Klebsiella pneumoniae* Strains Isolated From Neonates With Sepsis

**DOI:** 10.3389/fmed.2018.00225

**Published:** 2018-08-14

**Authors:** Khalit S. Khaertynov, Vladimir A. Anokhin, Albert A. Rizvanov, Yuriy N. Davidyuk, Dina R. Semyenova, Sergey A. Lubin, Natalia N. Skvortsova

**Affiliations:** ^1^Department of Children Infectious Diseases, Kazan State Medical University, Kazan, Russia; ^2^Institute of Fundamental Medicine and Biology, Kazan Federal University, Kazan, Russia; ^3^The Neonatal Intensive Care Unit, City Children's Clinical Hospital N1, Kazan, Russia; ^4^Republican Clinical Infectious Diseases Hospital, Kazan, Russia

**Keywords:** neonatal sepsis, *Klebsiella pneumoniae*, virulence, antibiotic resistance, extended-spectrum beta-lactamases

## Abstract

**Introduction:** *Klebsiella pneumoniae* is one of the most important infectious agents in neonates. There are “classic” and hypervirulent strains of *K. pneumoniae*. The “classic” non-virulent strain of *K. pneumoniae*, producing extended-spectrum beta-lactamases (ESBLs), is associated with nosocomial infections. Hypervirulent *K. pneumoniae* strains are associated with invasive infections in previously healthy adult people, and most of them exhibit antimicrobial susceptibility. The role of virulent strains of *K. pneumoniae* (including hv-KP) in neonatal infections is unknown. The aim of the study was the assessment of the impact of virulence factors and antibiotic resistance of *K. pneumoniae* strains on clinical features and outcomes of neonatal infection.

**Materials and Methods:** Two groups of infants were enrolled. The first group consisted of 10 neonates with sepsis caused by *K. pneumoniae*. The second group consisted of 10 neonates with urinary tract infection (UTI) caused by *K. pneumoniae*. We investigated the susceptibility of *K. pneumoniae* isolates to antibiotics, the ability of the microorganism to produce ESBL, and virulence factors, including the *rmpA* gene, aerobactin, and colibactin genes. In neonates with sepsis, we investigated *K. pneumoniae* isolates, which was taken from the blood, in neonates with UTI—from the urine.

**Results:** In neonates with sepsis testing of *K. pneumoniae* isolates for ESBL production was positive in 60% of cases, in neonates with UTI—in 40% of cases. All blood and urine ESBL producing *K. pneumoniae* isolates were resistant to ampicillins, including protected ones, and third-generation cephalosporins. At the same time, these isolates were sensitive to meropenem, amikacin, and ciprofloxacin. The *rmpA* gene was detected in four blood, and three urine *K. pneumoniae* isolates. In neonates with sepsis *rmpA* gene in two cases was detected in ESBL-producing *K. pneumoniae* isolates. They were infants with meningitis, and both cases were fatal. In the group of infants with UTI, the *rmpA* gene was detected only in *K. pneumoniae* isolates not producing ESBL. Aerobactin and colibactin genes were detected in two neonates with sepsis and in three neonates with UTI. In all cases, aerobactin and colibactin genes were detected only in *rmpA*-positive *K. pneumoniae* isolates. Out of three fatal outcomes, two cases were caused by hv-KP producing ESBL.

**Conclusion:** The prevalence of virulent strains of *K. pneumoniae* among neonates with sepsis and other neonatal infection is higher than we think. The most severe forms of neonatal sepsis with an unfavorable outcome in our study were due to virulent strains of *K. pneumoniae*.

## Introduction

*Klebsiella pneumoniae* is one of the leading causes of neonatal sepsis (NS) (1, 2). This microorganism accounts for NS in 4–9% of cases in developed countries, and 16–28% in developing countries (3–6). There are “classic” and hypervirulent strains of *K. pneumoniae* (7). “Classic” non-virulent *K. pneumoniae* (c-KP) strains are usually associated with pneumonia, urinary tract infection, nosocomial infections, and neonatal sepsis in immunocompromised patients (8). C-KP can cause NS outbreaks in hospitals and ICU (9, 10). C-KP strains have recently gained notoriety due to their propensity to acquire antimicrobial resistance. Within the last few decades, extended-spectrum β-lactamase (ESBL)-positive *K. pneumoniae* isolates have been recovered worldwide, especially in intensive care units (ICU) (11). The prevalence of ESBL-producing strains of *K. pneumoniae* is 23% in the USA, and up to 85–100% in some European countries (11). Hypervirulent strains of *K. pneumoniae* (hv-KP) were first recognized in Taiwan in the last twentieth century and caused liver abscesses, meningitis, and endophthalmitis in previously healthy adult patients (12–14). Currently, hv-KP strains are being spread in different parts of the world (15, 16). High virulence of hv-KP is associated mainly with enhanced capsule production, that can be triggered by a regulator of the mucoid phenotype (*rmpA*) gene and mucoviscosity-associated gene A (*magA*) (7, 17). In addition to c-KP and hv-KP, in recent years the third type of *K. pneumoniae* was detected, which characterized by a combination of antibiotic resistance and hypervirulence (16, 18, 19). The prevalence of antibiotic resistance in hv-KP isolates is rare compared with the high prevalence of antibiotic resistant c-KP isolates (20). A report in 2016 showed that in China 12.6% of hv-KP isolates from several invasive infections produced ESBL (21). The role of virulent strains of *K. pneumoniae* (including hv-KP) in neonatal infections is unknown. We hypothesized that virulent determinants of *K. pneumoniae* might affect clinical features and outcomes on neonatal infection. We assume that virulent strains of *K. pneumoniae* in neonates can lead to the development of the most severe forms of neonatal infection (purulent meningitis and sepsis with one or more foci of infection). C-KP in neonates is associated mainly with prematurity, the immaturity of the immune system, prolonged staying in the intensive care unit (ICU), mechanical ventilation, using intravenous or urinary catheters (1). Therefore, the main clinical manifestations of neonatal infection caused by c-KP can be pneumonia, urinary tract infection, and septicemia.

The aim of the study was the assessment of the impact of virulence factors and antibiotic resistance of *K. pneumoniae* strains on clinical features and outcomes of neonatal infection.

## Materials and methods

### Subjects

This study was conducted over a period of 18 months between May 2015 and October 2016. Two groups of neonates were enrolled. The first group consisted of 10 neonates with culture-proven sepsis, caused by *K. pneumoniae*. Neonates with sepsis were admitted to the Neonatal Intensive Care Unit (NICU) of the City Children's Clinical Hospital N1 in Kazan. During this period, the diagnosis of culture proven sepsis was established for 28 neonates admitted in NICU of this hospital. In 14 cases (50%), neonatal sepsis was caused by *Staphylococcus* spp., in 13 cases (46%)—by *K. pneumoniae*, and one case was due to *Candida* spp. Seven neonates have died. Most of them (six neonates, 86%) were neonates with sepsis caused by *K. pneumoniae*. In accordance with the Report of the Expert Meeting on Neonatal and Pediatric Sepsis (8 June 2010, EMA, London) (22), sepsis was defined as the presence of at least two clinical symptoms and at least two laboratory signs in presence of or as a result of a suspected or proven infection (positive blood culture). The clinical symptoms are (1) body temperature instability; (2) cardiovascular instability; (3) skin and subcutaneous lesions such as petechial rash or sclerema; (4) apnoea or increased oxygen requirement, or requirement for ventilation support; (5) feeding intolerance or abdominal distension; and (6) irritability, lethargy, or hypotonia. Its laboratory signs are (1) a white blood cell (WBC) count of <4 or >20 × 10^9^ cells/L; (2) an immature to total neutrophil ratio (I/T) of >0.2; (3) a platelet count of <100 × 10^9^/L; (4) C-reactive protein (CRP) levels of >15 mg/dL; (5) blood glucose values of >180 mg/dL or hypoglycemia (<40 mg/dL) confirmed at least twice; and (6) metabolic acidosis as characterized by a base excess (BE) of ≤10 mmol/L. Blood cultures of all 10 neonates included in the study were positive for *K. pneumoniae*. The second group (comparison group) consisted of 10 neonates with urinary tract infection (UTI). The criteria for inclusion of neonates in this group were leukocyturia (>10 leukocytes/mL) and bacteriuria (>10^5^ colony-forming units/mL). Four out of ten infants had a fever and were admitted to the hospital, another six were outpatient infants and had no any clinical symptoms. From the urine of all infants was isolated *K. pneumoniae*.

We studied the antibiotic susceptibility of *K. pneumoniae*, its ability to produce ESBL, and its virulence factors including *rmpA*, aerobactin, and colibactin genes. A distinctive feature of hv-KP is its hypermucoviscous phenotype.

The Ethics Committee of the City children's hospital approved this study, the written informed consent obtained from the parents following the principles authorized under this protocol (the Federal Law N323-FL dated on 21.11. 2011 “On Protection of Health of the Citizens in the Russian Federation”).

### CRP analysis

Serum CRP levels were evaluated with the use of the Randox Full Range CRP immunoturbidimetry assay (Randox Laboratories, Crumlin, Northern Ireland, UK), according to the manufacturer's recommendations.

### Bacterial isolates

Blood samples: 1 ml of blood was collected with a sterile syringe and mixed with 20 ml of a Brain-Heart Infusion broth (Conda Pronadisa, Spain). The mixture was incubated at 37°C for 7 days, streaked onto the surface of blood agar and MacConkey agar (Oxoid, UK) and incubated at 37°C for 24 h (23). Urine samples were collected in sterile containers. A loopful of the urine samples were streaked onto the surface of blood agar and MacConkey agar (Oxoid, UK) and incubated at 37°C for 24 h. All isolates were identified according to morphological and biochemical tests (Gram stain, capsule stain, motility test, indole production test, urease production test, Methyl Red test, Voges-Proskauer test) (24) and confirmed by matrix-assisted laser desorption-ionization time-of-flight mass spectrometry (Microflex, Bruker Daltonics, Bremen, Germany).

### Antibiotic susceptibility testing

The antibiotic susceptibility of *K. pneumoniae* isolates was determined by the Kirby–Bauer disk diffusion method according to Clinical and Laboratory Standards Institute guidelines (CLSI) (25). Suspension of each *K. pneumoniae* isolate was spread by sterile glass rods on the surface of *Mueller Hinton agar* (Oxoid, UK). Then antibiotic discs (BioRad, France) were placed onto the surface of the inoculated *Mueller Hinton agar* plate. The plate was then incubated at 35°C for 18 h. Antimicrobial susceptibility was determined by measuring the diameter of the inhibition zone according to CLSI (25).

### Test for extended-spectrum β-lactamase production

The *K. pneumoniae* isolates were evaluated for ESBLs by using a double-disk method according to CLSI (25). Amoxicillin/clavulanate discs were placed in the center of *Mueller Hinton* agar plate (Oxoid, UK). The disc of ceftazidime and cefotaxime were placed at the distance of 20 mm from the amoxicillin/clavulanic acid disc. The plates were incubated aerobically at 35°C for 18 h before the zone size recorded. A positive result was indicated when the inhibition zones around any of the cephalosporin discs were augmented in the direction of the disc containing clavulanic acid.

### Hypermucoviscousity testing

Single colonies, cultured on blood agar and MacConkey agar plates (Oxoid, UK), were obtained and studied for their ability to form viscous strings. The hypermucoviscousit*y* was determined by the formation of viscous strings of >5 mm length (14). Isolation of *K. pneumoniae* strains and microbiological studies of obtained isolates were performed in the bacteriological laboratory of the Republican Clinical Infectious Diseases Hospital.

### DNA extraction

Bacterial colonies were grown on the surface of blood agar and MacConkey agar, collected and suspended in 50–100 μl of sterile distilled water. Total DNA was extracted from suspended cells with the use of an extraction kit (“Litech,” Russia) according to the manufacturer's recommendations.

### Polymerase chain reaction (PCR) detection of virulence-associated genes

DNA samples were analyzed by using polymerase chain reaction (PCR). Primer sequences used to amplify fragments of the target genes are listed in Table 1. The reaction mix composition and PCR programs for amplification can be provided upon request. The amplicones were separated in 1.5% agarose gel and purified with the use of the GeneJET Gel Extraction Kit (Thermo Scientific, USA) according to the manufacturer's recommendations. The PCR-products were sequenced on a 3730 DNA Analyzer (Life technologies, USA) and compared with *K. pneumoniae* str. Kp52.145 plasmid II and chromosomal genome sequences from GenBank (Accession numbers FO834905.1 and FO834906.1, respectively) to confirm the target genes.

**Table 1:** Primers used for PCR.

**Target gene**	**Primer sequence (5′ → 3′)**	**Size of PCR-product, bp**	**References**
*rmpA*	Forward (ACGACTTTCAAGAGAAATGA)	418	(26)
*rmpA*	Reverse (CATAGATGTCATAATCACAC)		(26)
*rmpA*	Forward (CATAAGAGTATTGGTTGACAG)	462	(27)
*rmpA*	Reverse (CTTGCATGAGCCATCTTTCA)		(27)
*iroD*	Forward (GCATAGGCGGATACGAACAT)	531	(26)
*iroD*	Reverse (CACAGGGCAATTGCTTACCT)		(26)
*iroN*	Forward (CTGTCGGCATCGGTTTTATT)	556	(28)
*iroN*	Reverse (TGGCGTGTCGATTATTACCA)		(28)
*clbA*	Forward (ATGAGGATTGATATATTAATTGGAC)	579	(29)
*clbA*	Reverse (ATTCTGCCCATTTGACGAATG)		(29)
*clbB*	Forward (GATTTGGATACTGGCGATAACCG)	732	(29)
*clbB*	Reverse (CCATTTCCCGTTTGAGCACAC)		(29)

## Results

### Study subjects

#### First group

Out of 10 neonates with NS 5 were the term and 5 were preterm. Three preterm neonates were born earlier than 32 weeks of gestation, and another two ones—in the period from 32 to 36 weeks of gestation (Table 2). Two neonates were born with extremely low body weight (ELBW). Sepsis developed in five neonates on the first week of life, in three ones on the 2nd and 3rd weeks, respectively, and in two neonates on the 4th week. Sepsis was diagnosed in 5 neonates during their staying in a maternity ward, and in three ones (neonates with the ELBW)—in the NICU of the Children's Clinical Hospital. Two neonates fell ill at home.

**Table 2:** Clinical characteristics of patients (Me; interquartile range).

**Parameters**	**Neonatal sepsis group, *n* = 10**	**Neonates with UTI, *n* = 10**
Gestational age (weeks)	36 [31–40]	38 [38–39]
Birth weight (gram)	2,600 [1,350–3,400]	3,200 [3,000–3,510]
Gender (male), abs. (%)	6 (60)	8 (80)
Apgar score	7 [6–7]	8 [8–8]
Clinical manifestation, abs. (%):		
enterocolitis; - meningitis; - pneumonia and enterocolitis; - septicemia; - urinary tract infection	3 (30) 3 (30) 2 (20) 2 (20)	10 (100)
Mechanical ventilation, abs. (%)	8 (80)	
C-reactive protein (mg/dl)	2.5 [1.2–4.8]	0.15 [0.1–0.2]
White Blood Cells (×10^9^/L)	12.6 [3.4–22.2]	9.8 [8.7–11.2]
Platelets (×10^9^/L)	35.5 [20–46]	270 [240-300]
Died, abs. (%)	3 (30)	–

NS clinically manifested in purulent meningitis (*n* = 3), enterocolitis (*n* = 3), and pneumonia plus enterocolitis (*n* = 2). In two cases the disease was associated with septicemia. Three mothers of neonates with sepsis had colpitis during pregnancy. The patients received a comprehensive treatment, including antibiotics, infusion therapy, mechanical ventilation, and intravenous immunoglobulins. Three cases of neonatal sepsis were fatal; two neonates had purulent meningitis and one necrotizing enterocolitis. Other seven neonates with sepsis successfully recovered.

#### Second group

Out of 10 neonates with UTI eight babies were born term and two—preterm. Both preterm neonates were born at 35 weeks of gestation. UTI developed in four neonates on the 3rd week of life, in six neonates on the 4th week. Only four out of ten infants had clinical manifestations, the main of which were fever and irritability. In the remaining six infants, UTI was asymptomatic. Two mothers of neonates with UTI had colpitis during pregnancy, in one case—pyelonephritis. All neonates received antibiotic therapy based on the findings of microbiological sensitivity tests.

#### Antimicrobial susceptibility

In neonates with NS, all *K. pneumoniae* isolates were resistant to ampicillin, and six of them being resistant to all aminopenicillins (including protected ones), gentamycin and third-generation cephalosporins. At the same time, all *K. pneumoniae* isolates were sensitive to meropenem, amikacin, and ciprofloxacin (Table 3). Six isolates of *K. pneumoniae* produced ESBLs. In neonates with UTI, all *K. pneumoniae* isolates also were resistant to ampicillins. Six of them were resistant to gentamycin, four isolates—to protected aminopenicillins and third generation cephalosporins. All *K. pneumoniae* isolates were sensitive to meropenem, amikacin, and ciprofloxacin. Test for ESBL production was positive for 4 isolates of *K. pneumoniae*.

**Table 3:** Antibiotic susceptibility of *K.pneumoniae* isolates^*^.

***N***	**Clinical manifestation**	Antibiotic/mcg/disk^**^									
		**AX**	**AMC**	**CAZ**	**CTX**	**CRO**	**CP**	**C**	**MEM**	**AK**	**GN**
		**25**	**30**	**30**	**30**	**30**	**30**	**5**	**10**	**30**	**10**
**NEONATAL SEPSIS GROUP**											
1	Meningitis	R	S	S	S	S	S	S	S	S	S
2	Meningitis	R	R	R	R	R	R	S	S	S	R
3	Meningitis	R	R	R	R	R	R	S	S	S	R
4	Enterocolitis	R	S	S	S	S	S	S	S	S	S
5	Enterocolitis	R	R	R	R	R	R	S	S	S	R
6	Enterocolitis + Pneumonia	R	R	R	R	R	R	S	S	S	R
7	Enterocolitis + Pneumonia	R	R	R	R	R	R	S	S	S	R
8	Septicemia	R	S	S	S	S	S	S	S	S	S
9	Septicemia	R	S	S	S	S	S	S	S	S	S
10	Enterocolitis	R	R	R	R	R	R	S	S	S	R
**NEONATES WITH URINARY TRACT INFECTION**											
1	UTI	R	S	S	S	S	S	S	S	S	S
2	UTI	R	S	S	S	S	S	S	S	S	S
3	UTI	R	R	R	R	R	R	S	S	S	R
4	UTI	R	R	R	R	R	R	S	S	S	R
5	UTI	R	R	R	R	R	R	S	S	S	R
6	UTI	R	S	S	S	S	S	S	S	S	S
7	UTI	R	R	R	R	R	R	S	S	S	R
8	UTI	R	S	S	S	S	S	S	S	S	S
9	UTI	R	S	S	S	S	S	S	S	S	S
10	UTI	R	S	S	S	S	S	S	S	S	S

* *S, susceptible; R, resistant*.

** *AX, amoxicillin; AMC, amoxicillin/clavulanat; CAZ, ceftazidim; CTX, cefotaxim; CRO, ceftriaxon; CP, cefepime; C, ciprofloxacin; MEM, meropenem; AK, amikacin; GN, gentamycin*.

#### Virulence factors

In the neonatal sepsis group, the *rmpA* gene (the main virulence factor of *K. pneumoniae*) was detected in 4 cases (40%). The presence of the *rmpA* gene in *K. pneumoniae* was associated with the development of purulent meningitis (3 cases) and enterocolitis (1 case). Only full-term infants had meningitis. Two of four children with the *rmpA* gene in *K. pneumoniae* isolates died. The “string test” was positive if the colony of *K. pneumoniae* showed the presence of the *rmpA* gene only. Aerobactin and colibactin genes were detected in two *K. pneumoniae* isolates. Both aerobactin-producing isolates were positive for *iro D* and *iro N*. One of two colibactin-producing isolates was positive for *clb A* and *clb B*, another was positive for *clb B* only. Aerobactin and colibactin genes were detected only in *K. pneumoniae* isolates, taken from the neonates with purulent meningitis who had an unfavorable outcome (Table 4). The production of aerobactin and colibactin directly correlated with the presence of the *rmpA* gene. The aerobactin and colibactin genes were not detected in two *rmpA*-positive *K.pneumoniae* isolates. In one case it was an infant with meningitis, who had a favorable outcome, in the other case—an infant with enterocolitis.

**Table 4:** Virulence factors of *K. pneumoniae* isolate in neonates with sepsis and UTI^*^.

***N***	**Clinical manifestation**	**Outcome**	ESBL^**^	**rmpA**	**Aerobactin**		**Colibactin**	
					**iro D**	**iro N**	**clb A**	**clb B**
**NEONATAL SEPSIS GROUP**								
1	Meningitis	Recovered	−	+	−	−	−	−
2	Meningitis	Died	+	+	+	+	+	+
3	Meningitis	Died	+	+	+	+	+	
4	enterocolitis	Recovered	−	+	−	−	−	−
5	Enterocolitis	Recovered	+	−	−	−	−	−
6	Enterocolitis + Pneumonia	Recovered	+	−	−	−	−	−
7	Enterocolitis + Pneumonia	Recovered	+	−	−	−	−	−
8	Septicemia	Recovered	−	−	−	−	−	−
9	Septicemia	Recovered	−−	−	−	−	−	−
10	Enterocolitis	Died	+	−	−	−	−	−
**NEONATES WITH URINARY TRACT INFECTION**								
1	UTI	Recovered	−	+	+	+	+	+
2	UTI	Recovered	−	+	+	+	+	+
3	UTI	Recovered	+	−	−	−	−	−
4	UTI	Recovered	+	−	−	−	−	−
5	UTI	Recovered	+	−	−	−	−	−
6	UTI	Recovered	−	−	−	−	−	−
7	UTI	Recovered	+	−	−	−	−	−
8	UTI	Recovered	−	+	+	+	+	+
9	UTI	Recovered	−	−	−	−	−	−
10	UTI	Recovered	−	−	−	−	−	−

* *UTI, urinary tract infection*.

** *ESBL, extended-spectrum beta-lactamases*.

In neonates with UTI the *rmpA* gene, aerobactin, and colibactin genes were detected in 3 (30%) *K. pneumoniae* isolates. All aerobactin-producing isolates were positive for *iro D* and *iro N*. All colibactin-producing isolates were positive for *clb A* and *clb B*. The aerobactin and colibactin genes were not detected in *rmpA*-negaitive *K. pneumoniae* isolates.

The study of the impact of virulence factors of *K. pneumoniae* isolates on the clinical manifestations of neonatal infections revealed a high probability of the development of purulent meningitis (OR 9; CI 0.7–113) with an unfavorable outcome (OR 4.8; CI 0.3–65.7), but these differences were not statistically significant (Table 5).

**Table 5:** Comparison of clinical and laboratory data from neonates with and without virulent determinants.

**Variables, abs. (%)**	**With virulence (*n* = 7)**	**Without virulence (*n* = 13)**	OR, CI, p^*^
Gestational age, <38 weeks, abs. (%)	1 (14)	6 (46)	0.2; 0.1–2.1; 0.3
Birth weight, <3,000 g, abs. (%)	2 (28)	7 (55)	0.3; 0.05–2.4; 0.3
Gender (male), abs. (%)	3 (43)	3 (23)	2.5; 0.3–18; 0.6
Clinical manifestation, abs. (%):			
- enterocolitis; - meningitis; - pneumonia and enterocolitis; - septicemia; - UTI	1 (14) 3 (43) – – 3 (43)	2 (15) – 2 (15) – 2 (15) 7 (55)	0.9; 0.1–12.3; 1.0 9; 0.7–113; 0.08 0.9; 0.1–12; 1.0 0.9; 0.1–12; 1.0 0.6; 0.1–4; 0.6
Community-acquired, abs. (%)	5 (71)	7 (55)	2.1; 0.3–15; 0.6
C-reactive protein, >2,5 mg/dl, abs. (%)	3 (43)	2 (15)	4.1; 0.5–34.5; 0.2
White Blood Cells, >20 × 10^9^/L, abs. (%)	2 (28)	2 (15)	2.2; 0.2–20; 0.6
Platelets, <50 × 10^9^/L, abs. (%)	3 (43)	5 (38)	1.2; 0.2–7.7
Mechanical ventilation	4 (57)	4 (31)	4.4; 0.6–32; 0.3
Died, abs. (%)	2 (28)	1 (8)	4.8; 0.3–65.7; 0.2

* *OR, Odds ratio; CI, coincidence interval; p, Fisher's exact test*.

## Discussion

Numerous studies have shown that antibiotic resistance and virulence of microorganisms are significant risk factors for NS (30, 31). Antibiotic resistance of bacteria is the main cause of non-effective therapy of nosocomial infections and sepsis. The antibiotic resistance of *K. pneumoniae* strains is associated mainly with the production of ESBL. In 2017 the World Health Organization included ESBL-producing *K. pneumoniae* in the list of the most dangerous superbugs along with *Acinetobacter baumani* and *Pseudomonas aeruguinosa* (32). Neonates are most vulnerable to infection. The predisposing factors to Gram-negative sepsis in newborns are associated with prematurity and low birth weight, as well as a prolonged stay in ICU (33, 34). In our study, in the neonatal sepsis group, five neonates were preterm, and two of them were born with extremely low body weight (ELBW). Six blood isolates of *K. pneumoniae* strains (60%) produced ESBLs. In neonates with UTI, *K. pneumoniae* isolates produced ESBL in 40% of cases. Contamination of neonates with ESBL producing *K. pneumoniae* strains could occur in a maternity ward, or during prolonged staying in ICU. Testing for antibiotic resistance showed that all ESBL producing *K. pneumoniae* isolates (from blood and urine) were resistant to all aminopicillins and third-generation cephalosporins. At the same time, all ESBL producing *K. pneumoniae* isolates were sensitive to meropenem, amikacin, and ciprofloxacin. Obviously, that meropenem should be considered as priority antibiotic therapy in cases of neonatal infections caused by ESBL-producing strains of *K. pneumoniae*. All blood and urine *K. pneumoniae* isolate, not producing ESBL, were sensitive to protected aminopenicillins, third-generation cephalosporins, meropenem, amikacin, and ciprofloxacin. Therefore, in cases of neonatal infections caused by ESBL-not producing strains of *K. pneumoniae*, protected aminopenicillins and cephalosporins III should be used as the first line of antibiotic therapy. The early onset of antibiotic therapy is known to play a crucial role in the efficacy of sepsis therapy since its delay increases the risk of death (35). However, clinical symptoms and laboratory signs of NS are not specific, and its early identification is a rather difficult task. The involvement of gastrointestinal tract may indirectly indicate the development of infection caused by Gram-negative bacteria, as the intestine is the main site of their colonization. Among laboratory signs of sepsis associated with gram-negative bacteria, a number of researchers designate common development of thrombocytopenia (36). In our study, all 10 neonates with sepsis had thrombocytopenia, which was severe (<50 × 10^9^/L) in 8 ones. Five infants had enterocolitis. The most severe forms of sepsis in the infants studied, as well as its adverse outcomes, were associated with meningitis. It is worthy to note that neonatal meningitis occurred only in full-term infants, two of them were discharged home from maternity wards and fell ill on the second week of life. When analyzing this situation, we tend to associate the development of sepsis in neonates with their staying in maternity wards, because most *K. pneumoniae* isolates were ESBL-producers. This also can be supported by the fact that sepsis caused by *K. pneumoniae* occurred in six neonates who were in the same hospital during the same period. Unfortunately, neither medical staff of the maternity houses nor the mothers of these neonates were examined for *K. pneumoniae* carriage, so the above assumption cannot be confirmed. Despite the well-known role of ESBL-producing *K. pneumoniae* strains in the development of late onset of NS, it is also evident that the virulence factors of *K. pneumoniae* play an essential role in the development of the most severe forms of NS. The factors that are implicated in the virulence of *K. pneumoniae* strains include capsular polysaccharides, lipopolysaccharides, fimbrial adhesins, and siderophores (37, 38). The hypervirulent phenotype of *K. pneumoniae* is associated with the presence of the *rmpA* gene (14). Hv-KP causes the most severe, invasive forms of infection in previously healthy adults (liver abscesses, meningitis, and endophthalmitis) (12–14). Unlike cv-KP, hv-KP strains are more resistant to the complement and phagocytosis (38, 39). The mechanisms providing such stability are diverse and include protection against opsonization and phagocytosis by macrophages and neutrophils due to the presence of a thick capsule in *K. pneumoniae* and the blockade of TLR, in particular, TLR4, which inhibits the expression of interleukin-8 (38). Commonly, high virulence is typical for *K. pneumoniae* strains that cause community-acquired infections, and these microorganisms are usually sensitive to the majority of external antimicrobial factors. At the same time, recent studies demonstrate a new trend: hv-KP have acquired the main property of nosocomial microorganisms—antibacterial resistance. The impact of the hv-KP strain on the clinical features of neonatal infection is unknown. In our study, the *rmpA* gene was found in *K. pneumoniae* isolates in both groups of neonates, with NS and UTI. The detection of the *rmpA* gene in *K. pneumoniae* strains isolated from neonates with sepsis in three of four cases was associated with purulent meningitis. The detection of the *rmpA* gene in neonates with UTI was unexpected for us. Obviously, that other virulence factors, in particular, lipopolysaccharide (LPS) and siderophores, are necessary to ensure the virulence of *K. pneumoniae*. LPS protects against humoral defenses and activate immune system (20). Lipid A of LPS is a ligand for TLR4 (20). O-antigen of LPS protects against complement (20). Strains of *K. pneumoniae* that contain a full-length O-antigen (“smooth LPS”) are resistant to complement-mediating killing, while those with truncated or absent of O chains (“rough LPS”) are susceptible to complement-mediating killing, even in the presence of capsule (40). The lack of O-antigen *K. pneumoniae* less virulent. Detection of *the rmpA* gene in *K. pneumoniae* isolates in neonates with UTI indicates that the prevalence of virulent *K. pneumoniae* strains may be higher than we think. The prevalence of hv-KP in Russian Federation is unknown. The prevalence of hv-KP is very high in Asia (up to 90%) (41), in other regions of the world, it is less. In Spain and Canada, the prevalence of hv-KP was estimated at 5.4 and 8.2%, respectively (42, 43). In our study, two cases were caused by ESBL-producing *K. pneumoniae* isolates which were containing the *rmpA* gene. They were infants with meningitis, and both cases were fatal. In the group of infants with UTI, the *rmpA* gene was detected only in *K. pneumoniae* isolates not producing ESBL. Despite the small number of neonates included in this study, it is obvious that the acquisition of multidrug resistance by virulent strains of *K. pneumoniae* can worsen the course and prognosis of the disease. Siderophores—aerobactin, enterobactin, yersiniabactin, and colibactin are most important virulent factors that promote *K. pneumoniae* growth and survival in an infected host (7, 38). Enterobactin expression is ubiquitous among c-KP and hv-KP (44, 45). But aerobactin is expressed by 6% of c-KP and 93–100% of hv-KP isolates (44, 45). Siderophores are small, high-affinity iron chelating molecules secreted by a wide variety of microorganisms that are critical for virulence in many Gram-negative bacteria (7, 20). Iron is known to be essential for bacterial growth and replication (46). Access to iron molecules is an important factor that provides invasive properties of *K. pneumoniae* in an infection in immunocompetent people (46). Hv-KP strains produce siderophore molecules in a larger number and in their more active form than c-KP do (46). It has been shown that the development of invasive forms of the disease is associated with the production of several siderophores by *K. pneumoniae* strains (47). In our study, *K. pneumoniae* strains producing siderophores were detected in two neonates with sepsis and in three neonates with UTI. In all cases, aerobactin and colibactin genes were detected only in *rmpA*-positive *K. pneumoniae* isolates. However, fatal outcomes were noted only in cases caused by virulent strains of KP. In spite of the association between development of purulent meningitis and an unfavorable outcome in neonates infected mainly by virulent KP strains, we were not able to confirm this situation by statistical methods. Probably, this is due to a small number of newborns with purulent meningitis, which rarely occurs in this age group. We could not confirm the initial hypothesis that the development only the most severe forms of neonatal infection (purulent meningitis) is associated with virulent KP strains. UTI also can be caused by virulent strains of *K. pneumoniae*. Obviously, the role of virulent strains of *K. pneumoniae* will be increased in the future.

## Conclusion

The prevalence of virulent strains of *K. pneumoniae* among neonates with sepsis and other neonatal infection is higher than we think. The risk of development of severe forms of neonatal infection can be associated with virulence factors. The most severe forms of neonatal sepsis with an unfavorable outcome in our study were due to virulent strains of *K. pneumoniae*.

## Author contributions

KK, VA, and AR: design of the study, writing the manuscript. YD: DNA extraction of *K. pneumoniae* strains with subsequent genotyping by PCR method to determine virulence factors. DS: collection of clinical data. SL: collection and interpretation of clinical data. NS: isolation of *K. pneumoniae* colonies, determination of their antibiotic resistance and ability to produce of extended spectrum β-lactamase.

### Conflict of interest statement

The authors declare that the research was conducted in the absence of any commercial or financial relationships that could be construed as a potential conflict of interest.
